# What explains the link between childhood ADHD and adolescent depression? Investigating the role of peer relationships and academic attainment

**DOI:** 10.1007/s00787-019-01463-w

**Published:** 2020-01-13

**Authors:** Victoria Powell, Lucy Riglin, Gemma Hammerton, Olga Eyre, Joanna Martin, Richard Anney, Anita Thapar, Frances Rice

**Affiliations:** 1 MRC Centre for Neuropsychiatric Genetics and Genomics, Division of Psychological Medicine and Clinical Neurosciences, Cardiff University, Cardiff, UK; 2 Population Health Sciences, University of Bristol, Bristol, UK

**Keywords:** Depression, ADHD, Peer relationships, Academic attainment, ALSPAC

## Abstract

There is increasing evidence that childhood Attention-Deficit Hyperactivity Disorder (ADHD) elevates risk of later depression, but the mechanisms behind this association are unclear. We investigated the relationship between childhood ADHD symptoms and late-adolescent depressive symptoms in a population cohort, and examined whether academic attainment and peer problems mediated this association. ALSPAC (Avon Longitudinal Study of Parents and Children) is an ongoing prospective longitudinal population-based UK cohort that has collected data since September 1990. 2950 individuals with data on parent-reported ADHD symptoms in childhood (7.5 years) and self-reported depressive symptoms in late adolescence (17.5 years) were included in analyses. 2161 individuals with additional data at age 16 years on parent-reported peer problems as an indicator of peer relationships and formal examination results (General Certificate of Secondary Education; GCSE) as an indicator of academic attainment were included in mediation analyses. Childhood ADHD symptoms were associated with higher depressive symptoms (*b* = 0.49, SE = 0.11, *p* < 0.001) and an increased odds of clinically significant depressive symptoms in adolescence (OR = 1.27, 95% CI 1.15–1.41, *p* < 0.001). The association with depressive symptoms was mediated in part by peer problems and academic attainment which accounted for 14.68% and 20.13% of the total effect, respectively. Childhood ADHD is associated with increased risk of later depression. The relationship is mediated in part by peer relationships and academic attainment. This highlights peer relationships and academic attainment as potential targets of depression prevention and intervention in those with ADHD. Future research should investigate which aspects of peer relationships are important in conferring later risk for depression.

**Electronic supplementary material:**

The online version of this article (10.1007/s00787-019-01463-w) contains supplementary material, which is available to authorized users.

## Introduction

Major Depressive Disorder (MDD) is a leading cause of disability worldwide [1]. The incidence of MDD rises markedly in adolescence and peaks in early adulthood [2, 3]. Depression has a complex aetiology [4] and various psychiatric disorders in childhood have been shown to increase risk of subsequent depression [5]. Clinical long-term follow-up studies of children with Attention-Deficit Hyperactivity Disorder (ADHD) show substantially elevated rates of subsequent depression in this group [6–9]. One longitudinal population-based study reported that 50% of those meeting clinical criteria for ADHD in adolescence had MDD or an anxiety disorder at a follow-up assessment (range of 2–11 years) compared to 35% in those without ADHD [10]. As well as being viewed as a diagnostic category, there is good evidence that ADHD behaves as a continuously distributed risk in the general population [11]. Thus, population-based studies are useful for examining longitudinal links between ADHD symptoms and depression. While there is evidence of a prospective relationship between ADHD and depression across development, the reasons for this association are unclear. Further studies are needed to understand how childhood ADHD increases depression risk as this can help to inform interventions to support young people with ADHD. For those with clinical diagnoses of ADHD, treating core ADHD symptoms is important and may reduce concurrent and subsequent depression risk [12]. However, treatment trials showing the long-term benefits of ADHD treatment on depression are lacking. In addition, preliminary evidence shows that those with depression and ADHD may be at higher risk of antidepressant resistance than those with depression alone [13]. Thus, additional factors that contribute to depression outcomes in those with ADHD need consideration, as these could give insight into additional intervention targets.

**Fig. 1:**
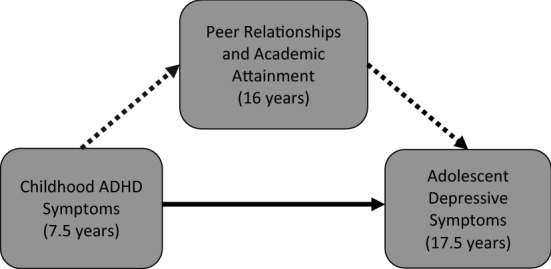
The hypothesized mediation model was that part of the association between childhood ADHD and adolescent depression would be explained by peer relationships and academic attainment

Two possible factors linking ADHD and depression are difficulties in peer relationships and academic attainment. Difficulties with peer relationships have been shown to be important in depression aetiology [14–16] and children with ADHD often have difficulties with peers [17, 18]. Academic attainment in examinations and other aspects of educational performance including motivation, the ability to function in the classroom, and general cognitive ability have also been associated with ADHD [19, 20] and depression [16, 21–24]. A number of theories suggest that the child’s sense of competency or failure with peers and in academic domains is important in conferring later depression risk [25–27]. Therefore, difficulties with peer relationships and academic attainment could link ADHD with subsequent depression by acting as mediators (Fig. 1). However, a meta-analytic review of the association between ADHD and depression noted that there are few mechanistic studies [6]. Two studies to date have tested mediation using longitudinal data with time-lags between exposure, mediator, and outcome [28, 29], an important part of testing mediation [30]. One focused on social functioning, social acceptance, and child perceptions of academic functioning as mediators [28]. The other examined peer victimization and asked children who they disliked and who they bullied in their class [29]. The first included 472 participants in a sample selected to over-represent the children of depressed mothers [28]. Mothers completed the ‘attention problems’ subscale of the Child Behavior Checklist (CBCL) [31] at age 5 to index childhood ADHD. In a multiple mediator model, a latent variable capturing social functioning and social acceptance mediated the association between attention problems at age 5 and depressive symptoms 15 years later. Although general school functioning as measured by ‘academic stress’ was correlated with attention problems and depressive symptoms in the study, Humphreys and colleagues found that academic stress did not act as a mediator in their multiple mediator model. The second study was conducted in a longitudinal population sample of 728 participants aged approximately 13 at baseline [29]. A peer nomination approach was used where children were asked to report who in their class they disliked and who they bullied. This mediated 7% of the relationship between the CBCL attention problems subscale (a combination of self, parent, and teacher reports) and depression approximately 5 years later. Therefore, these two longitudinal studies examining different potential mediators highlight the need for studies examining additional aspects of peer relationships and the role of academic attainment in the longitudinal relationship of ADHD and depression. More generally, there is a clear need for studies spanning childhood and adolescence to investigate the mechanisms underlying the association between ADHD and depression. In the present study, we tested whether peer relationships and academic attainment mediated the association between childhood ADHD and adolescent depressive symptomology in a large prospective population study spanning 10 years (Fig. 1). We hypothesized that our findings would confirm a prospective association between childhood ADHD symptoms and adolescent depressive symptoms, and show that peer relationships and academic attainment contribute to this association.

## Methods

### Sample

This sample was derived from the Avon Longitudinal Study of Parents and Children (ALSPAC) (https://www.alspac.bris.ac.uk), a large prospective birth cohort in South-West England. Pregnant women in Bristol with a due date between April 1991 and December 1992 were approached to participate. This resulted in 14,541 pregnancies enrolled in the study and 13,988 children alive at 1 year. The children’s development has been followed regularly since birth largely via questionnaires and face-to-face assessments. The methodology and sample are described elsewhere, but the population is generally representative of UK children [32, 33]. Please note that the ALSPAC website contains details of all available data through a searchable data dictionary (https://www.bristol.ac.uk/alspac/researchers/our-data/). This study is based on a primary sample of 2950 participants for whom data were available on childhood ADHD symptoms, depressive symptoms in late adolescence, and covariates (socioeconomic status, maternal age at birth, sex and childhood emotional problems). Mediation analyses included 2161 participants where data were additionally available on peer problems and academic attainment at 16 years.

### Measures

#### Childhood ADHD symptoms

The main exposure variable was mother-rated DSM-IV (Diagnostic and Statistical Manual of Mental Disorders, 4th edition) [34] ADHD symptoms at 7 years and 7 months measured with the Development and Well-Being Assessment (DAWBA) [35]. The DAWBA is a well-validated, reliable tool which can be used to derive DSM-IV diagnoses in children and adolescents via computer algorithms and clinical raters. Symptoms of ADHD include inattention, for example “often forgetful in daily activities”, hyperactivity such as “often fidgets with hands or feet”, and impulsivity such as “often interrupts or intrudes on others” that are inconsistent with developmental level, and have been present for 6 months or more, with some symptoms causing impairment in multiple domains before age 7. Symptoms were classed as present if mothers reported them occurring in their child “a little” or “a lot” more than in other children to create a count ranging from 0 to 18. DSM-IV [34] diagnosis of ADHD derived from the DAWBA was used in a sensitivity analysis.

#### Late-adolescent depression

Continuous and binary depression variables were used as the outcome in this study to give an indication of the impact of ADHD on depressive symptoms and on the odds of reaching a clinical cut-point for depression. For the continuous outcome, ‘depressive symptom score’ was indicated by the self-rated Short Moods and Feelings Questionnaire (SMFQ) at 17 years and 6 months. The SMFQ is a 13-item questionnaire designed to cover core depressive symptomology with a three-point response scale for each question of ‘Not true’ (0), ‘Somewhat true’ (1), or ‘True’ (2) summed to generate a maximum score of 26 [36]. For the binary outcome, those scoring ≥ 12 on the SMFQ were classed as having ‘clinically significant depressive symptoms’ as recommended previously [37]. The SMFQ is a reliable, valid measure of adolescent depression [37], with high sensitivity and specificity for detecting DSM-IV [38] and ICD-10 [39] MDD diagnoses [40, 41].

#### Mediator variables

Mediator variables were measured at age 16 years. This ensured a time lag between exposure (age 7.5), mediator (age 16) and outcome (age 17.5) as recommended for mediation analysis [30].

##### Peer relationships

The 5-item Peer Problem subscale of the mother-completed Strengths and Difficulties Questionnaire (SDQ) at 16 years was used [42]. Items such as ‘Teenager has at least one good friend’ and ‘Teenager is generally liked by others’ were rated with responses of ‘Not true’ (2), ‘Sometimes true’ (1), and ‘Certainly true’ (0), with higher scores indicating more peer problems. Items were summed to generate a total score (maximum = 10). The SDQ is a well-validated, reliable behavioural screening tool in young people [42].

##### Academic attainment

Academic attainment was assessed by performance in formal examinations at the end of secondary school at 16 years (General Certificate of Secondary Education; GCSE examinations). GCSEs are graded from A* (highest grade achievable) to U (lowest grade achievable). A total GCSE and equivalents point score was calculated by summing individual point scores for each GCSE and equivalent qualification grade achieved (A* being equivalent to 58 points, A to 52, B to 46, etc.) [43].

#### Confounding variables

Analyses were adjusted for mother’s socioeconomic status according to occupation and maternal age at birth to account for sociodemographic factors associated with depression [44]. These were available from mother-reported questionnaires completed during pregnancy or the early years of the study child’s life. Analyses were additionally adjusted for the child’s sex. Sex and sociodemographic variables could potentially confound all three paths between variables tested in the mediation analyses (Fig. 1). Thus, all analyses presented are adjusted for both.

### Data analysis

#### Association between ADHD and depression

Childhood ADHD symptoms were standardised, so that a unit increase was equivalent to a standard deviation unit increase. Linear regression was used to examine the association between standardized childhood ADHD symptoms and continuous depressive symptom score at 17.5 years. Logistic regression was used to examine the association between standardised childhood ADHD symptoms and depression assessed using the binary SMFQ clinical cut-point at 17.5 years. To examine whether sex influenced the association between ADHD symptoms and depression, an interaction term of ADHD symptoms and sex was regressed against depressive symptoms. The Wald test was then used to test whether the model with the interaction term was significantly different to the model without the interaction term. Regressions were also repeated using an exposure variable of ADHD diagnosis.

#### Mediation by peer relationships and academic attainment

Peer problems and GCSE result scores at 16 years were tested as mediators of the association between childhood ADHD symptoms and adolescent depressive symptoms in two single mediator models. A ‘potential outcomes’ causal mediation framework was used with STATA commands ‘medeff’ and ‘medsens’ [45, 46]. Medeff conducts mediation analyses using Monte Carlo simulation whilst allowing for interaction of exposure and mediator on the outcome. Medsens considers mediation models’ sensitivity to potential confounding by unobserved confounders of the mediator–outcome relationship. This mediation method was selected because the traditional mediation methods can be subject to biases resulting from not considering potential exposure–mediator interaction or unobserved confounding of the mediator–outcome association [47]. Confidence intervals for indirect effects were estimated using a non-parametric bootstrapping approach with 10,000 replications [48]. Reported statistics are Pure Natural Direct Effect (PNDE), Total Natural Indirect Effect (TNIE), and percentage of the total effect that was mediated. PNDE is the direct (unmediated) effect of the exposure on the outcome when the mediator takes the value it would take in the absence of the exposure. TNIE captures the mediated effect of the exposure on the outcome that operates by changing the mediator [49]. In addition, the effect of simultaneously estimating mediation by peer relationships and academic attainment was tested in a multiple mediator model using Structural Equation Modelling (SEM) [50]. SEM uses a conceptual model and a series of regression-like equations to capture complex relationships within a network of variables, which is expressed as a path diagram. SEM does not have the ‘potential outcomes’ advantages of considering exposure–mediator interaction or mediator–outcome confounding, but is suited to testing more complex mediation models.

#### Sensitivity analyses

As previous work has found victimization to mediate the association between ADHD symptoms and subsequent depression [29], we additionally tested whether observations of mediation effects by peer problems were driven by victimisation. To do this, the peer problem mediation analysis was repeated with the item “young person is picked on or bullied by other young people” removed from the Peer Problems score of the SDQ [42].

Three sensitivity tests were conducted to check for the effect of timing of variables. Use of mediator data collected at an earlier time point than depression data does not ensure that these mediators actually preceded depressive symptoms. Thus, mediation analyses were repeated adjusting for depressive symptoms at 14 years (prior to the measurement of mediators) to account for depression occurring earlier in adolescence (Supplement 1). Furthermore, although academic attainment data were not available at an earlier age than 16 years, we repeated the peer relationship mediation analysis using peer problem and depression data collected at earlier time points. We used SDQ peer problems data collected at 9.5 years (prior to the typical age of onset of depression) and SMFQ depressive symptoms at age 13 years (Supplement 2). In addition, we checked whether observed associations between ADHD and adolescent depression were due to ‘pre-existing’ childhood emotional problems occurring approximately contemporaneously with ADHD. This could confound the direct association between ADHD and depressive symptoms. Therefore, emotional problems as indicated by the SDQ emotional problems subscale at 8 years (with a score of ≥ 5 classed as ‘abnormal’) [42] were adjusted for in an additional regression. However, it is possible that childhood emotional problems may be on the causal pathway between ADHD and depression. Therefore, it was not included as a covariate in the fully adjusted regressions.

#### Missing data

To address the possibility of bias due to non-random missing data, all analyses were repeated with Inverse Probability Weighting (IPW) applied. IPW is a reliable technique for handling missing data, particularly in longitudinal studies where participants can have missing values on multiple variables [51]. Weights were generated based on predictors of missingness from the analysis sample (Supplement 3), as conducted previously in ALSPAC [52]. To investigate predictors of missingness, childhood ADHD symptoms, sex, and sociodemographic variables were individually regressed against missingness from mediator or outcome data (Supplement 4).

## Results

In the 2950 participants (1298 males and 1652 females) with complete data, 0.47% (*n* = 14) met the criteria for DSM-IV ADHD diagnosis at 7.5 years. DSM-IV ADHD symptoms (mean = 3.58, standard deviation = 4.55) were used to define the primary ADHD exposure variable. 17.32% (*n* = 511) met the clinical cut-point for depression at 17.5 years (mean symptom score = 6.45, standard deviation = 5.18). As expected, the majority of those with ADHD were male (85.71%) and the majority of those reaching the binary cut-point for clinically significant depressive symptoms were female (66.73%). Table 1 shows the correlations of analysis variables.

**Table 1:** Correlation matrix of analysis variables

Variable	ADHD symptoms	Peer problems	GCSE results	Depressive symptoms
ADHD symptoms	1	0.21*	− 0.24*	0.08*
Peer problems		1	− 0.11*	0.11*
GCSE results			1	− 0.08*
Depressive symptoms				1

Pearson correlations of mediation variables were conducted on complete cases for exposure, mediators, outcome, and confounders (*n* = 2161)

*ADHD* attention-deficit hyperactivity disorder, *GCSE* general certificate of secondary education

*Correlation significant at *p* < 0.001

### Association between childhood ADHD and adolescent depression

Standardised childhood ADHD symptoms predicted the continuous outcome of adolescent depressive symptom score (*b* = 0.49, SE = 0.11, *p* < 0.001). There was no significant interaction between ADHD symptoms and sex in predicting depressive symptom score (Wald test: *F* = 0.99, d.f. = (1, 2946), *p* = 0.32), though the relationship was slightly stronger in females (*b* = 0.59, SE = 0.17, *p* < 0.001) than males (*b* = 0.40, SE = 0.13, *p* = 0.002).

Childhood ADHD symptoms predicted the binary outcome of clinically significant depressive symptoms at age 17.5 years (OR = 1.27, 95% CI = 1.15–1.41, *p* < 0.001). This remained the case when adjusting for socioeconomic factors, sex and childhood emotional problems (Table 2). When examining sex differences, there was no significant interaction between ADHD symptoms and sex in predicting clinically significant depressive symptoms (Wald test: *X*^2^ = 2.02, d.f. = (1), *p* = 0.16), though the association was slightly stronger in females (OR = 1.36, 95% CI = 1.18–1.56, *p* < 0.001) than males (OR = 1.18, 95% CI = 1.01–1.37, *p* = 0.04). Clinical diagnosis of ADHD (*n* = 14: 12 males and 2 females) was also associated with increased odds of clinically significant depressive symptoms at 17.5 years (OR = 4.49, 95% CI = 1.53–13.19, *p* = 0.006).

**Table 2:** Association between childhood ADHD and late-adolescent depression

	Unadjusted OR, 95% CI, *p*	Adjusted for emotional problems at 8 years OR, 95% CI, *p*	Adjusted for sociodemographic variables OR, 95% CI, *p*	Adjusted for sex OR, 95% CI, *p*	Fully adjusted (adjusted for sociodemographic variables and sex) OR, 95% CI, *p*
Overall (*n* = 2950)	1.21, 1.10–1.34, < 0.001	1.19, 1.08–1.31, 0.001	1.20, 1.09–1.33, < 0.001	1.29, 1.16–1.42, < 0.001	1.27, 1.15–1.41, < 0.001
Males (*n* = 1298)	1.18, 1.01–1.38, 0.03	1.13, 0.97–1.33, 0.12	1.18, 1.01–1.37, 0.04		
Females (*n* = 1652)	1.37, 1.20–1.57, < 0.001	1.36, 1.19–1.56, < 0.001	1.36, 1.18–1.56, < 0.001		

*ADHD* attention-deficit hyperactivity disorder, *OR* odds ratio, *CI* confidence interval

### The role of peer relationships and academic attainment in the association between ADHD and depression

Peer relationships at 16 years mediated the association between childhood ADHD symptoms and late-adolescent depressive symptoms, accounting for 14.68% of the total effect (Table 3). Peer problems remained a mediator when the item tapping victimization—“picked on or bullied by other young people”—was removed (Total Natural Indirect Effect: *b* = 0.07, 95% CI = 0.01–0.14), accounting for 12.50% of the association.

Academic attainment mediated the association between childhood ADHD symptoms and late-adolescent depressive symptoms, accounting for 20.13% of the total effect (Table 3).

**Table 3:** Mediation of the association between childhood ADHD and adolescent depression

Mediator (measured at 16 years)	Pure natural direct effect b (95% CI)	Total natural indirect effect b (95% CI)	Proportion of total effect mediated % (95% CI)
Peer problems	0.45 (0.19–0.70)	0.08 (0.02–0.15)	14.68 (9.97–28.78)
GCSE results	0.41 (0.15–0.66)	0.10 (0.01–0.21)	20.13 (13.89–41.81)

The exposure levels being compared in these analyses were mean ADHD symptoms and 1 Standard Deviation above this. Significant mediators are indicated by confidence intervals of ‘Total Natural Indirect Effect’ not containing zero. *n* = 2161

*ADHD* attention-deficit hyperactivity disorder, *GCSE* general certificate of secondary education, *b* unstandardized beta, *CI* bootstrapped confidence interval

When testing peer relationships and academic attainment simultaneously in a multiple mediator SEM model, mediated pathways between ADHD and depressive symptoms via peer relationships and academic attainment revealed a similar pattern of results (Supplement 5).

One of the advantages of the potential outcomes approach to mediation is that it allowed testing of potential unobserved confounding of the mediator–depression relationship. The ‘medsens’ test indicated that the product of observed variance in mediator and outcome that would need to be explained by an unmeasured confounder for mediation effects to disappear ranged from approximately 0.005–0.008 (Supplement 6). To aid interpretation of this coefficient, we compared it with the estimate for a measured confounder—socioeconomic status of mother. The estimate for socioeconomic status of mother on mediation by academic attainment was 0.0002. The coefficient for unobserved confounding required to eliminate the observed mediated effects is, therefore, considerably larger than that present for the measured confounder of socioeconomic status. Thus, it seems unlikely that unobserved confounding would account for the observed results.

### Inverse probability weighting (IPW)

Results remained very similar when IPW was applied to analyses. Childhood ADHD symptoms remained associated with the continuous late-adolescent depressive symptom score (*b* = 0.55, SE = 0.12, *p* < 0.001) and with the binary measure of clinically significant depressive symptoms in late adolescence (OR = 1.29, 95% CI = 1.16–1.44, *p* < 0.001). Mediation results with IPW applied are shown in Supplement 7.

## Discussion

This study investigated the prospective association between childhood ADHD symptoms and late-adolescent depressive symptoms over a 10-year period in a population cohort and evaluated potential mediation by peer relationships and academic attainment. Results confirmed an association between childhood ADHD symptoms and adolescent depressive symptoms in line with previous findings [6–10], supporting a longitudinal relationship between ADHD and depression in the general population.

Mediation results demonstrated that difficulties with peer relationships and academic attainment at 16 years contribute to the pathway from childhood ADHD symptoms to adolescent depressive symptoms as we hypothesized (Fig. 1). This finding is in line with previously proposed models which suggest that young people who struggle in their school and social lives are more likely to experience feelings of failure and rejection, which leave them more vulnerable to depression and emotional problems [26, 27]. The present results help reconcile previous uncertainties in the literature as to whether peer and academic-related factors contribute to the association of ADHD and depression, stemming from a lack of studies investigating longitudinal mediation of this association [6]. Peer problems explained 14.68% of the total effect between ADHD and depressive symptoms. This aligns with previous findings of a latent variable capturing social functioning and social acceptance mediating an association between attention problems (as measured by the attention problems subscale of the CBCL [31]) and depressive symptoms in a sample selected to over-represent the children of depressed mothers [28]. We also show that peer relationships may play an important part in the pathway from ADHD to depression and that this is not driven entirely by the effect of being bullied, which has been evidenced to contribute to the longitudinal association of ADHD and depression [29]. In our study, peer problems still mediated 12.50% of the total effect when the “young person is picked on or bullied by other young people” item of the scale was removed. This highlights that additional aspects of peer relationships such as having good friendships, feeling liked by others, and playing or socialising with others may also be important protective factors for depression in those with ADHD. Indeed, it is important to consider that different aspects of peer relationships may have different effects in those with ADHD, which might impact on the most appropriate choice of target for depression interventions. For instance, there is strong evidence that peer victimisation increases later depression risk [53, 54]. In addition, good quality peer relationships can support young people at elevated familial risk for depression and have been shown to be associated with mental health resilience in this group [55].

Academic attainment also mediated the link between ADHD and depression in our study with GCSE results explaining 20.13% of the association between childhood ADHD and adolescent depressive symptoms. This finding contrasts somewhat with results of a longitudinal mediation study that found that child perceptions of academic stress did not mediate the association of attention problems and depressive symptoms [28]. However, differences between the two studies may account for this. In particular, the academic mediator differed in that the present study assessed academic attainment by performance in formal public examinations (GCSE results), while Humphreys and colleagues used a life stress interview at age 15 to assess perceptions of stress and functioning at school. The current study focused on the two measured mediators of academic attainment and peer relationships, whereas Humphrey’s and colleagues used mediation models comprising of multiple latent variables capturing functioning in various domains. Due to these study differences, it is difficult to make any comparisons of the mediation results. However, it is interesting to consider whether different aspects of school life have distinctive effects in those with elevated ADHD symptoms. While the other study found that general functioning in school was not a mediator of the association of attention problems and depressive symptoms [28], we found that academic attainment (GCSE exam results) mediated the association of ADHD and depressive symptoms. This suggests that academic attainment may be an important target within school life for depression intervention in those with ADHD. Nonetheless, comparison of results from these contrasting study designs should be interpreted with caution.

Those with ADHD may struggle with academic attainment for various reasons, including difficulties with formal classroom learning where sustained attention, self-control, emotion modulation, and adherence to rules are frequently required. In essence, mediation analysis involves specifying a potentially causal sequence where an antecedent influences a mediator which influences an outcome. Thus, although IQ may affect academic attainment [56], we did not test for a possible mediating effect of IQ between childhood ADHD and late-adolescent depression because of the assumptions this would imply about the temporal ordering of variables. We focused on academic attainment as a mediator as it was assessed at a time point between childhood ADHD and late-adolescent depression where it could influence later depression. Academic attainment may also potentially be more amenable to intervention when compared to IQ. Furthermore, the characteristics required for formal classroom learning such as self-control have been shown to predict academic attainment measured by school grades more strongly than IQ [57]. A systematic review of school-based non-pharmacological interventions for ADHD found that the expectations of the classroom were not a good “fit” for those with ADHD, which had a negative impact on academic performance and peer interaction [58]. Repeated infringements of classroom expectations resulting in lowered academic attainment and less positive peer interactions may lead to feelings of failure and isolation, thus increasing risk for developing depression [26, 27]. Overall, our findings suggest that reduced ability to form and maintain friendships and perform well academically may drive part of the development of adolescent depression in those with childhood ADHD in the general population.

It is worth noting that the peer relationships and academic attainment mediators may represent person-effects on the environment, vice versa, or both [59]. As ADHD and depression share genetic liability [60, 61], shared genetic liability with the mediators cannot be ruled out as an explanation of results. Also of note is the observation that young people with ADHD have been found to under-report their depressive symptoms [62], which may lead to an underestimation of associations.

Limitations of this study include the finite number of mediators investigated, although mediators studied were hypothesis-driven. Mediators were tested independently as we elected to use potential-outcome mediation with the ‘medeff’ STATA command, which does not allow simultaneous estimation. The potential-outcome method overcomes limitations of traditional mediation approaches, including consideration of exposure–mediator interaction and unobserved confounding of the mediator-outcome association [47]. However, to check that the results did not differ when testing the mediators simultaneously, we also tested a multiple mediator model using SEM [50]. In this model, mediated pathways via peer relationships and academic attainment were both significant (Supplement 5). Assessment of the mediators at an earlier time point than depressive symptoms does not guarantee that these factors preceded depressive symptoms. However, regressions adjusted for earlier childhood emotional problems showed that childhood ADHD was still associated with depression at 17.5 years (Table 1). Mediation analyses adjusted for depressive symptoms at 14 years—prior to the measurement of peer relationships or academic attainment—also remained very similar (Supplement 1). However, this sensitivity check should be interpreted with caution, as depression at 14 would almost certainly be affected by the exposure and thus act as an intermediate confounder. Furthermore, although academic attainment data were only available at 16 years, we repeated the peer problem mediation analysis using data collected at earlier time points. We found that peer problems at age 9.5 years (prior to the typical age of onset of depression) still mediated the association between ADHD symptoms at age 7.5 years and depressive symptoms at age 13 years, accounting for over 20% of the total relationship (Supplement 2). Attrition is an issue in ALSPAC as with many longitudinal population cohorts [63]. Missingness at 17.5 years was predicted by childhood ADHD symptoms, meaning that those with ADHD may be under-represented. Indeed, ADHD diagnosis prevalence in this study (0.47%) was lower than reported in the UK population of children (1.5%) [64]. However, this would likely result in attenuation of associations if there was an effect on results. Inclusion of variables predicting missingness from depression data (socioeconomic status, maternal age at birth and sex) as confounders in analyses helped address bias that may be caused by missing data [65]. To investigate impact of further bias arising from missing data, we repeated analyses with Inverse Probability Weights applied. Results remained very similar, suggesting that the impact of missing data was minimal (Supplement 7) [51].

This study benefited from longitudinal data spanning 10 years, allowing the mediation of the relationship between ADHD and depression to be investigated across development from childhood to adolescence. Adolescence is an important stage to study due to its association with increased depression levels [2, 3]. Longitudinal data help to avoid potential misclassification of individuals as unaffected by depression that can occur when long-term follow-up to late adolescence is not possible. Longitudinal data allowed time-lags between measurement of exposure, mediator, and outcome—an important part of investigating developmental pathways with mediation analyses [30]. Use of a large prospective population sample avoided the selection bias that can result from including only those at the severe end of the disorder group, as is the case in previous clinical studies [6].

### Implications

Due to the increased risk of clinically significant depressive symptoms in children with ADHD, it is important to monitor this group for depression. Monitoring children with ADHD may allow early identification and treatment of depression. ADHD symptoms were associated with depressive symptoms partly via peer relationships (even when accounting for potential effects of bullying) and academic attainment. This indicates that the development of interventions targeting the impact of children’s ADHD on their peer relationships and academic attainment may have the added benefit of reducing depression risk, in addition to treating core ADHD symptoms. For instance, a number of existing psychosocial interventions available for those with ADHD that target social skills and academic skills [66–68] may have the potential to reduce depression risk, though this needs to be formally tested.

For future research, a greater understanding of the specific components of peer relationship difficulties in those with ADHD that confer depression risk would allow further insight into the most appropriate targets for intervention.

## Conclusions

We show in a longitudinal population cohort that childhood ADHD symptomology is associated with increased risk of clinically significant depressive symptoms in late adolescence. This adds to a growing body of research highlighting the need for depression monitoring in young people with ADHD. We found that the association is mediated in part by peer problems and academic attainment. This highlights these areas as potential targets for depression prevention and intervention in children with ADHD.

## Electronic supplementary material

Below is the link to the electronic supplementary material.

Supplementary file1 (DOCX 75 kb)
