# Supplementary material for: What explains the link between childhood ADHD and adolescent depression? Investigating the role of peer relationships and academic attainment
Source: Eur Child Adolesc Psychiatry. 2020 Jan 13;29(11):1581–91. doi: 10.1007/s00787-019-01463-w (PMC7595988; doi:10.1007/s00787-019-01463-w)
Supplement: Supplementary file 1 — Supplementary file1 (DOCX 75 kb) [file 787_2019_1463_MOESM1_ESM.docx]

**Supplementary Materials**

**Supplement 1: Mediation Results Adjusted for Depressive Symptoms Prior to Measurement of Mediators**

*Mediation of the Association between Childhood ADHD and Late Adolescent Depression Adjusted for Depression at 14 years old*

| Mediator  (Measured at 16 years) | Pure Natural Direct Effect  b (95% CI) | Total Natural Indirect Effect  b (95% CI) | Proportion of Total Effect Mediated  % (95% CI) |
| --- | --- | --- | --- |
| Peer problems | 0.47 (0.22-0.71) | 0.09 (0.02-0.17) | 16.35 (11.44-29.36) |
| GCSE results | 0.44 (0.19-0.70) | 0.11 (0.01-0.21) | 19.04 (13.46-36.62) |

Two individual potential outcomes causal mediation analyses each adjusted for sex, maternal age at birth and socioeconomic status were repeated with adjustment for the additional covariate of depressive symptoms at 14 years old. This was to account for the effect of depression prior to the measurement of the mediators which could affect peer relationships and GCSE results. The exposure levels being compared in these analyses are mean ADHD symptoms and 1 Standard Deviation above this. The mediation allowed for an interaction between exposure and mediator and was conducted on complete cases for exposure, mediators, outcome and confounders (n=2161). Interpretation of results remained the same. Significant mediators are indicated by confidence intervals of ‘Total Natural Indirect Effect’ not containing zero. ADHD=Attention-Deficit Hyperactivity Disorder; GCSE=General Certificate of Secondary Education; b=unstandardized beta; CI=Confidence Interval.

**Supplement 2: Peer Problems Mediation Result at an Earlier Time Point**

*Mediation of the Association between ADHD at 7.5 years and Depression at 13 years old by Peer Problems at 9.5 years old*

| Mediator  (Measured at 9.5 years) | Pure Natural Direct Effect  b (95% CI) | Total Natural Indirect Effect  b (95% CI) | Proportion of Total Effect Mediated  % (95% CI) |
| --- | --- | --- | --- |
| Peer problems | 0.22 (0.19-0.25) | 0.07 (0.05-0.08) | 22.83 (20.16-26.04) |

The peer problems individual potential outcomes causal mediation analyses adjusted for sex, maternal age at birth and socioeconomic status was repeated using data collected at earlier time points. This was done to check whether peer problems still mediated the association between ADHD and depression when it was measured at a time point prior to the typical age of onset of depression. The exposure levels being compared in these analyses are mean ADHD symptoms and 1 Standard Deviation above this. The mediation allowed for an interaction between exposure and mediator and was conducted on complete cases for exposure, mediators, outcome and confounders (n=4330). Interpretation of results remained the same. Significant mediators are indicated by confidence intervals of ‘Total Natural Indirect Effect’ not containing zero. ADHD=Attention-Deficit Hyperactivity Disorder; b=unstandardized beta; CI=Confidence Interval.

**Supplement 3: Inverse Probability Weighting**

Inverse Probability Weighting (IPW) was used to address potential bias caused by non-random missing data in our sample. It involves weighting the analysis sample by the inverse probability of being missing. IPW can be particularly useful in addressing missingness in samples with blocks of missing data (Seaman & White, 2013). This is often the case in ALSPAC where missingness from a group of variables occurs due to non-participation in a clinic assessment, for example. Based on the attrition analyses documented in Supplement 4, in those with data available on ADHD at 7 years, variables measured at early time points in the ALSPAC cohort predicting missingness from depression data at 17.5 years and mediator data at 16 years were examined and formed two missingness models – one predicting missingness from the outcome and another predicting missingness from both the outcome and mediators – from which two weights were created. Minimal missing data on these predictors were singly imputed as the modal value (all predictors had <14% of values missing). The Hosmer-Lemeshow test showed no indication of poor fit for the outcome missingness model (Hosmer-Lemeshow χ2(8)=4.11, p=0.85) or for the outcome and mediators missingness model (Hosmer-Lemeshow χ2(8)=11.93, p=0.15). Weights ranged from 1.36 to 23.45. Regressions and mediation analyses were re-run with the respective IPW weights applied to address potential bias caused by missing data. Results were very similar to the unweighted results (Supplement 7).

**Supplement 4: Attrition Analyses**

*Prediction of Missingness from Analysis Sample at 17.5 Years*

| Predictor Variable | OR | 95% CI | P-value |
| --- | --- | --- | --- |
| ADHD symptoms at 7 years 7 months | 1.22 | 1.16-1.29 | <0.001 |
| Sex (1=male, 2=female)*** | 0.66 | 0.60-0.73 | <0.001 |
| Month of birth** | 1.00 | 0.99-1.02 | 0.65 |
| Socioeconomic status based on occupation of mother is ‘unskilled’*** | 1.19 | 1.03-1.37 | 0.02 |
| Maternal age at delivery of child*** | 0.95 | 0.94-0.96 | <0.001 |
| Number of children mother had before study child*** | 1.11 | 1.05-1.17 | <0.001 |
| Mother’s highest educational qualification is a degree*** | 0.63 | 0.55-0.71 | <0.001 |
| Mother owns a home during pregnancy*** | 0.42 | 0.36-0.49 | <0.001 |
| Mother had financial problems during pregnancy*** | 1.50 | 1.25-1.79 | <0.001 |
| Mother smoked during pregnancy*** | 1.77 | 1.51-2.08 | <0.001 |
| Mother’s social support score during pregnancy*** | 0.97 | 0.96-0.99 | <0.001 |
| Mother has a partner when child is 8 months old* | 0.54 | 0.39-0.75 | <0.001 |
| Mother ever had severe depression (reported during pregnancy)*** | 1.44 | 1.17-1.78 | 0.001 |
| Child’s mood score at 24 months old (from the Carey Toddler Temperament Scale)* | 1.01 | 1.00-1.02 | 0.22 |

Within those with ADHD symptom data at 7 years and 7 months (n=8182), logistic regressions between numerous predictor variables and being missing from the regression or mediation analysis sample were conducted to establish predictors of attrition in this study. Results shown here use missingness from mediation analysis as the outcome. * indicates this variable was used as a predictor in the missingness model used to generate Inverse Probability Weights (IPWs) for regression analyses as described in Supplement 3. ** indicates this variable was used to generate IPWs for mediation analyses. ***indicates this variable was used for both. ADHD=Attention-Deficit Hyperactivity Disorder; OR=Odds Ratio; CI=Confidence Interval.

**Supplement 5: Testing Mediators Simultaneously in Multiple Mediator Structural Equation Model**

**A**

ADHD symptoms

Depressive symptoms

Academic attainment

.34*

-.43*

-.21*

ADHD symptoms

Depressive symptoms

Peer relationships

.32*

.51*

.22*

**B**

**C**

ADHD symptoms

Depressive symptoms

Peer relationships

Academic attainment

.24

-.40*

.49*

-.21*

.22*

A sensitivity analysis to check that peer relationships and academic attainment still mediated the association of ADHD and depressive symptoms when tested simultaneously (Figure C) as opposed to individually (Figures A and B) was conducted using Structural Equation Modelling (SEM). This test was conducted on complete cases for exposure, mediators, outcome and confounders (n=2161). When both mediators were entered simultaneously, the direct association between ADHD and depressive symptoms became non-significant (p=0.06). Both mediated pathways via peer relationships and academic attainment remained significant at p<0.007. Beta coefficients are shown on the path arrows. ADHD=Attention-Deficit Hyperactivity Disorder.

**Supplement 6: Mediation Sensitivity Results**

*Sensitivity Analysis of Mediators of the Association between ADHD and Depression*

| Mediator | Rho at which ACME = 0 | R^2^_M~R^2^_Y~  at which ACME = 0 | R^2^_M*R^2^_Y*  at which ACME = 0 |
| --- | --- | --- | --- |
| Peer problems at 16 years | 0.09 or greater | 0.008 | 0.009 |
| GCSE results at 16 years | -0.07 or lower | 0.005 | 0.006 |

Sensitivity analysis to check for the effect of confounding of the association between mediator and outcome for each mediation analysis was conducted on complete cases for exposure, mediators, outcome and confounders (n=2161) using the ‘medsens’ package. ACME is the Average Causal Mediation Effect. ‘Rho at which ACME = 0’ is the correlation between residuals of mediator and outcome variable (as an indication of confounding of the mediator-confounder association) that would be needed for an observed mediation effect to disappear. ‘R^2^_M~R^2^_Y~ at which ACME=0’ is the product of how much of the observed variance in the mediator and in the outcome would need be explained by unobserved confounders for an observed mediation effect to disappear. ’R^2^_M*R^2^_Y* at which ACME = 0’ is the product of how much previously unexplained variance in the mediator and in the outcome would need to be explained by unobserved confounders for the mediation effect to disappear. For example, for the observed mediation of the association between ADHD and depression via peer problems to disappear, unobserved confounders would need to explain 9% of observed variance in mediator and 9% of observed variance in the outcome (the product of which is 0.008), with a Rho of 0.09 or greater for mediator-outcome confounding. As long as Rho is lower than 0.09, the mediation effect will still be observed. GCSE=General Certificate of Secondary Education.

**Supplement 7: Inverse Probability Weighted Mediation Results**

*Mediators of the Association between Childhood ADHD and Adolescent Depression with IPW Weights Applied*

| Mediator  (Measured at 16 years) | Pure Natural Direct Effect  b (95% CI) | Total Natural Indirect Effect  b (95% CI) | Proportion of Total Effect Mediated  % (95% CI) |
| --- | --- | --- | --- |
| Peer problems | 0.58 (0.29-0.87) | 0.09 (0.02-0.18) | 13.62 (9.59-23.92) |
| GCSE results | 0.54 (0.24-0.83) | 0.13 (-0.002-0.27) | 19.00 (13.62-34.35) |

Two individual potential outcomes causal mediation analyses each adjusted for sex, maternal age at birth and socioeconomic status were repeated with Inverse Probability Weights (IPW) applied to examine potential bias due to missing data on results of whether peer problems and GCSE results mediated the association between ADHD and depression. The exposure levels being compared in these analyses are mean ADHD symptoms and 1 Standard Deviation above this. Each mediation allowed for an interaction between exposure and mediator and was conducted on complete cases for exposure, mediators, outcome and confounders (n=2161). Results remained very similar with IPW weights applied. Significant mediators are indicated by confidence intervals of ‘Total Natural Indirect Effect’ not containing zero. ADHD=Attention-Deficit Hyperactivity Disorder; GCSE=General Certificate of Secondary Education; b=unstandardized beta; CI=Confidence Interval.
